# Identification of a truncated form of methionine sulfoxide reductase a expressed in mouse embryonic stem cells

**DOI:** 10.1186/1423-0127-18-46

**Published:** 2011-06-22

**Authors:** Pingping Jia, Chi Zhang, Yuanyuan Jia, Keith A Webster, Xupei Huang, Andrei A Kochegarov, Sharon L Lemanski, Larry F Lemanski

**Affiliations:** 1 Project to Cure Paralysis, University of Miami Miller School of Medicine, Miami, FL 33136, USA; 2 Department of Molecular and Cellular Pharmacology, University of Miami, Miller School of Medicine, Miami, FL 33431, USA; 3 Department of Biomedical Science, Florida Atlantic University; Boca Raton, FL 33101, USA; 4 Department of Anatomy and Cell Biology and The Cardiovascular Research Center, School of Medicine, Temple University, Philadelphia, Pennsylvania 19140, USA; 5 Department of Biological and Environmental Sciences, Texas A&M University-Commerce, Commerce, TX 75429-3011, USA

## Abstract

**Background:**

Methionine Sulfoxide Reductase A (MsrA), an enzyme in the Msr gene family, is important in the cellular anti-oxidative stress defense mechanism. It acts by reducing the oxidized methionine sulfoxide in proteins back to sulfide and by reducing the cellular level of reactive oxygen species. MsrA, the only enzyme in the Msr gene family that can reduce the S-form epimers of methionine sulfoxide, has been located in different cellular compartments including mitochondria, cytosol and nuclei of various cell lines.

**Methods:**

In the present study, we have isolated a truncated form of the MsrA transcript from cultured mouse embryonic stem cells and performed eGFP fusion protein expression, confocal microscopy and real time RT-PCR studies.

**Results:**

Results show a different expression response of this truncated transcript to oxygen deprivation and reoxygenation treatments in stem cells, compared to the longer full length form. In addition, a different subcellular localization pattern was noted with most of the eGFP fusion protein detected in the cytosol.

**Conclusion:**

One possibility for the existence of a truncated form of the MsrA transcripts could be that with a smaller protein size, yet retaining a GCWFG action site, this protein might have easier access to oxidize methionine residues on proteins than the longer form of the MsrA protein, thus having an evolutionary selection advantage. This research opens the door for further study on the role and function of the truncated MsrA embryonic mouse stem cells.

## Background

Free radical damage of cellular components, including proteins, has long been recognized in physiological aging and disease conditions. One of the cellular defense mechanisms to reduce the oxidized residues in proteins, and thus restore their functions as well as reduce oxidative stress, relies on the methionine sulfoxide reductase (msr) family genes, of which three MsrB genes (MsrB1, B2 and B3), have been identified in mammals [1-7]. Only one MsA gene has been found in mammals [1-7]. While both MsrA and MsrBs conduct the redox reactions with a similar chemical reaction mechanism, MsrBs can convert methionine-R-oxide (R-MetO) back to methionine while MsrA reduces methionine-S-oxide (S-MetO), respectively[4,8]. MsrBs localize at different cellular compartments: 1. MsrB1 is a cytosolic and nuclear protein; and 2. MsrB2 is targeted to mitochondria. Human MsrB3 gives rise to two forms generated by alternative first exon splicing, which are targeted to the endoplasmic reticulum (ER) and mitochondria [4] of the cell.

Although only a single MsrA gene is found in mammals, the corresponding protein is found to localize in multiple cellular compartments [9]. Further studies on human MsrA gene structures have identified two distinct putative promoters that generate three transcripts. The main MSRA transcript (MsrA1) was translated into the longest protein which localizes in mitochondria. MsrA2 and 3 originate from a second promoter and target the cytosol and nuclei [10,11]. More recent studies from rat smooth muscle cells revealed two novel splice forms: MsrA2a and MsrA2b [12]. The alternative splicing event occurred at the level of the second exon with MsrA2a coding a functional isoform. It seems that both alternative promoters and alternative splicing contribute to the variety of MsrA isoforms that are responsible for methionine sulfoxide reduction in different cellular compartments.

To date, most of the studies on MsrA isoforms are focused on the 5' terminus where a mitochondrial signal peptide is alternatively presented in different isoforms dictating whether or not the protein products are localized in the mitochondria [10,12,13]. Although there is evidence that transcripts of MsrA from alternative splicings at the 3' end of the MsrA gene are present in the mammalian EST database, due to the concern that these transcripts might not translate into enzymatically active protein products, no detailed studies to date have been reported on these transcripts [13].

We have consistently found a MsrA transcript from alternative splicing at the 3' end, skipping exon 5, thus producing a shorter isoform with a presumably truncated protein product containing the conserved catalytic active site in cultured mouse embryonic stem cells. Due to the importance of anti-oxidative stress mechanisms in stem cells, we have performed studies on this isoform in its expression pattern in normal culture conditions and its response to oxygen depletion/reoxygenation conditions in mouse embryonic stem cells.

## Methods

### Mouse embryonic stem cell culture

The mouse embryonic stem (MES) cells (CCE-24) were routinely grown on 0.1% gelatin-coated dishes in Dubecco's Modified Eagle's medium (DMEM) containing 15% heat-inactivated fetal bovine serum (catalog # 10100, Invitrogen, Carlsbad, CA), 10 ng/ml human leukemia inhibitory factor (LIF) (LIF2010, Millipore, Billerica, MA), and monothioglycerol (Sigma, St. Louis, Mo.) at 4.5 × 10-4 M [14]. Cells were grown on tissue culture plates coated with 0.1% gelatin (Sigma, St. Louis, WA) and routinely split every two days at 1:4 to 1:10 and immunostained for stem cell specific markers SSEA-1 (Mab4301) and SSEA-4 (Mab4304, Millipore, Billerica, MA) to ensure no differentiation. Only cells within the 20th passage were used.

### Anoxia/reoxygenation treatment

The anoxic treatment of mouse embryonic stem cells was performed by incubating the cells in an anaerobic chamber (Sheldon Manufacturing Inc., Cornelius, OR) supplied with 90% nitrogen gas, 5% hydrogen gas and 5% carbon dioxide at 37°C. Cells were removed after selected periods of treatment and incubated again in a regular cell culture incubator at 37°C for designated times.

Cloning of the truncated form of MsrA cDNA and construction of MsrA-truncated-eGFP fusion expression plasmid followed methods routine in our laboratory and recently published [15].

MsrA cDNA was amplified from the total RNA extracted from cultured embryonic stem cells using the primer pairs of: MsrA-for: 5'-cctggctgcggaggtggagaaac and MsrA-rev: 5'-atggccatcgggcaggaaactcc. The 744bp DNA band was gel purified and ligated into pGEM-T easy vector (Promega, WI). After sequencing to confirm gene sequences and rule out mutations, PCR was performed again using the following primer pairs to amplify the truncated form of MsrA and introduce a BamHI cutting site at the 3' end (MsrA-for: 5'-cctggctgcggaggtggagaaac and MsrA-BamHI-rev: 5'-tggggccaaggatccgctttgaaagaacc). The amplicons were gel purified and ligated into pEGFP-C vectors (Invitrogen, CA) to construct the truncated MsrA-eGFP expression plasmid. The truncated MsrA cDNA in the final plasmid was sequenced again to confirm the open reading frame.

### Confocal microscopy

After the MsrA-truncated-eGFP fusion expression plasmid was transfected into cells for three to five days and after confirmation of the GFP fluorescence signal by epi-fluorescent microscopy, 500 nM Mitotracker (Molecular Probe (Invitrogen), Carlsbad, CA) was diluted with complete culture medium and added to the cells. The cells were incubated for 30 minutes and checked under epi-fluorescence microscopy to confirm that they were well stained by the dye. The Mitotracker medium was discarded. Cells were washed with PBS and fixed in 1% Formaldhyde in PBS for 15 minutes at room temperature. Cells were rinsed with PBS twice and incubated in a 1/10000 dilution solution of DAPI (10 mg/ml) in PBS for 15-20 minutes. Cells were then mounted in the mounting solution Pro-Long (Molecular Probe, Carlsbad, CA), air-dried for 2 days in the dark, and coverslips were sealed to the slides with fingernail polish. Confocal microscopy was carried out using a Carl Zeiss confocal microscope at the University of Miami Diabetes Research Institute. Five micron Z-series were scanned for each sample.

### Real time RT-PCR

Real time RT-PCR experiments were performed according to our published procedures [16,17]. The primer sequences used in these studies were as follows:

MsrA-long form-for: 5'-TCTGGGTCTTGAAAGGAGTGTA;

MsrA-long-form-rev: 5'-AGGTATTGCTGGTGGTAGTCTTC; Amplicon size: 395bp.

MsrA-truncated-for: 5'-CGACCCGACCCAAGGTTCTTTCA;

MsrA-truncated-rev: 5'-GCCATCGGGCAGGAAACTCCAG; Amplicon size:168bp

β-actin-for: 5'-CCACTGCCGCATCCTCTTCCTC;

β-actin-for: 5'-CAGCAATGCCTGGGTACATGGTG; Amplicon size: 249bp.

## Results and Discussion

### Evidence of the existence of a smaller form of MsrA in mouse embryonic stem cells

**Figure 1:**
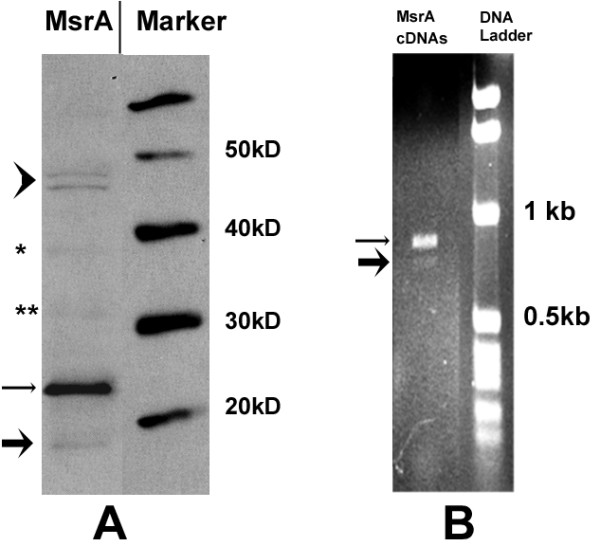
**Western blotting experiment using MsrA antibody reveals a smaller MsrA protein in cultured mouse embryonic stem cells**. A. Thick arrow: the smaller form (~16 kD); thin arrow: the large form (~23kD); arrowhead: possible dimers from the long form MsrA proteins; *: possible heterodimers from a large form and a shorter form protein; **: possible homodimers from two shorter form proteins. B. Agarose gel electrophoresis of the amplified cDNA of MsrA showing a smaller band. PCR bands: long form: 857bp (thin arrow); short form: 744bp (thick arrow).

From our previous studies on MsrA[15], we have consistently found that there is a noticeable protein band with a molecular weight (MW) of about 16 kD from Western blotting experiments using anti-MsrA antibody (Figure 1A, thick arrow). The hybridization signal is very low, usually becoming evident on the X-ray film with the longer form of MsrA bands overexposed (Figure 1A, thin arrow). There are also protein bands with molecular weights of ~46~48 kD indicating homo-dimers from MsrA long form proteins (Figure 1A, arrow head), even with protein samples thoroughly treated by reducing agents before SDS-PAGE, consistent with findings from other laboratories [10]. On the film, there is also a band with a molecular weight of 39 kD, possibly hetero-dimers formed by a long form protein of MsrA (MW: 23 kD) and a smaller form of MsrA protein (MW 16 kD) (Figure 1A, *). In addition, a faint band with a molecular weight of 32 kD, possibly the homo-dimers formed by two molecules of the smaller form of MsrA proteins is observable (Figure 1A, ** ). The 32kD and 39kD bands cannot readily be explained by the dimerization from cytosol isoforms of MsrA which are 19-20kD in size [10,13]. To confirm the existence of this smaller form of protein, we have carried out RT-PCR using total RNA extracted from mouse embryonic stem cells to amplify the full cDNA. The forward and reverse primers were designed based on the 5'-UTR and 3'-UTR of the known full length MsrA cDNA respectively (Genebank#: NM_026322.3 ). RT-PCR products were loaded onto a 1% agarose gel for electrophoresis; clearly two bands are visible on the gel with the smaller one (Figure 1B, thick arrow) showing only about 1/20 of the intensity and being about 100bp smaller than the larger band (Figure 1B, thin arrow).

### Cloning of the cDNA of the truncated form of MsrA

**Figure 2:**
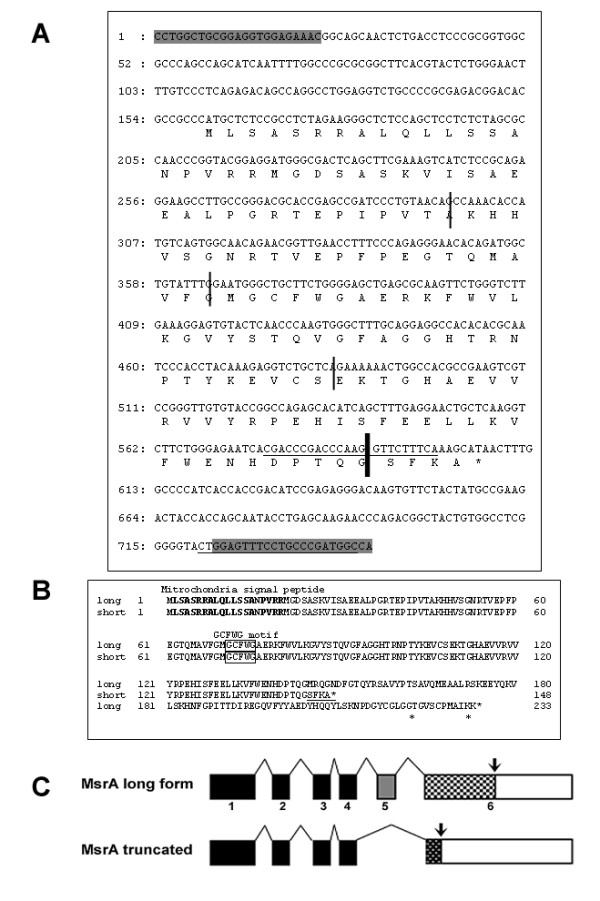
**The cDNA sequence of the truncated form of MsrA with the predicted open reading frame**. Thin lines indicate exon-exon junctions. A. The thick line points to the junction of exon 4 and 6 skipping exon 5 in the truncated form but is included in the long form of MsrA. Shaded sequences are the two primers used to amplify the whole cDNA. Sequences underlined show the primer pairs for real time PCR to specifically amplify the truncated form with the forward primer designed on the junction of exon 4 and 6. B. The truncated form contains 148 amino acids with a mitochondrial signal peptide at the N-terminus and a GCFWG motif but no c-terminal cysteines. C. Schematic illustration of exon compositions for the long form and truncated MsrA cDNA. Arrows point to the translation stop sites.

**Figure 3:**
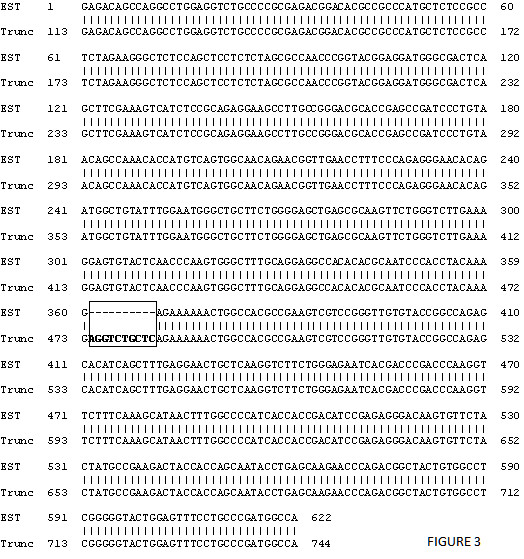
**Comparison between truncated form of MsrA cDNA (Trunc) and a mouse EST sequence from a kidney cDNA library in Genbank (EST)**. The EST sequence lacks ten nucleotides comparing with the truncated cDNA which is shown in the box. The same area is also shown in a box in Figure 2A, which is located at the very end of the third exon.

The RT-PCR products of the smaller band were recovered from agarose gels and ligated to the pGEM-T-easy vector (Invitrogen, CA). After DNA sequencing, the smaller form sequence was aligned and compared to the full length cDNA from the Genebank. A deletion of the fifth exon (113bp) was found in the smaller form which ends up with a frame shift in the sixth exon directly attached to the fourth that generated a new premature stop codon (Figure 2A, 2B). The total length of the truncated form protein is 148 amino acids, compared to the full length protein which has 233 amino acids, both containing a mitochondrial signal peptide at the N-terminus (Figure 2C). The truncated form still retains the GCFWG functional motif (catalytic active site) but contains neither of the two cysteines at the c-terminus (Figure 2B). Due to the functional importance of the two c-terminal cysteines in the redox reaction, it is reasonable to believe that the enzyme activity for methionine sulfoxide reduction will decrease dramatically, which needs to be confirmed by the studies on purified proteins translated from this truncated template. It is interesting that we have also identified a virtually identical mouse EST sequence from a kidney cDNA library in Genbank (Genbank ID: BG970953.1) with the same intron splicing pattern as the truncated form of MsrA cloned from embryonic stem cells, indicating this isoform might not be stem cell specific. The comparison between the EST sequence and truncated MsrA is illustrated in Figure 3. Except for missing ten nucleotides at the end of the third exon (boxes in Figure 3 and Figure 2A), the EST sequence showed 100% identity to the truncated cDNA from the 113th nucleotide (at the 5' UTR) to the very end of truncated form of the Msr cDNA.

### Confocal microscopy reveals different subcellular localizations for the truncated MsrA protein compared to the full length using eGFP fusion constructs

**Figure 4:**
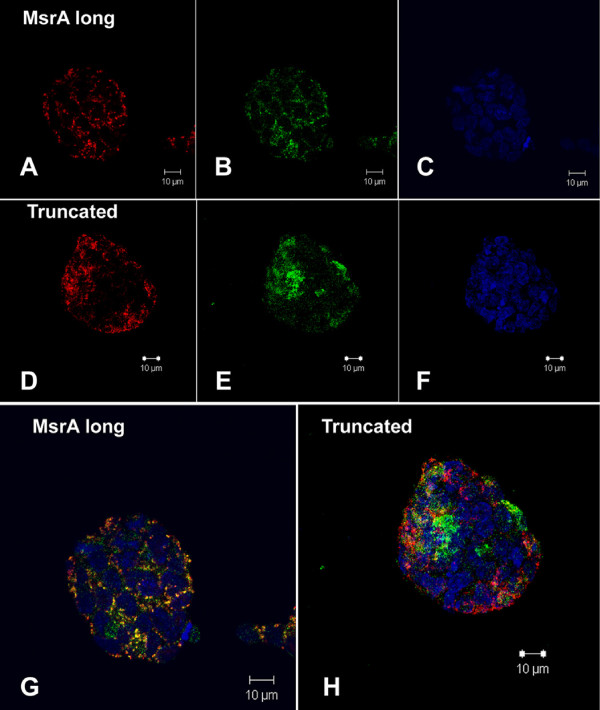
**Confocal microscopy on the long form MsrA-eGFP (A,B, C,G) and truncated MsrA-eGFP (D,E,F,H) transfected mouse embryonic stem cells**. A,D: mitochondria stained by propidium iodide; B,E: green fluorescence showing GFP tags; C,F: nuclei stained with DAPI; G: overlapped image from A, B and C, but with higher magnification; H: image overlapped from D, E and F; Bars: 10 μm.

**Figure 5:**
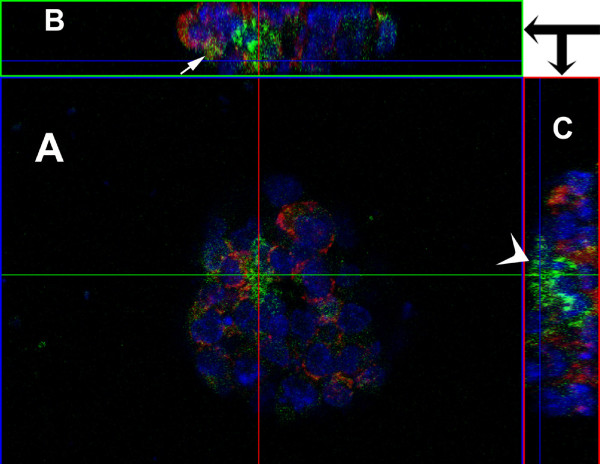
**Confocal microscopy of a single colony of mouse embryonic stem cells**. A: top view of a single slice of scanning showing most eGFP signals are not overlapping with mitochondria or nuclei. B: single slice scanning from upper-side view; J-2: single slice scanning from right-side view. The arrow in B indicates some overlapping signal of truncated Msr-eGFP and mitochondria. The arrowhead in C points to the area where the truncated Msr-eGFP signals are not overlapping with mitochondria, but mainly in cytosol. The crosspoint of the horizontal green line and vertical red line points to the area we are observing. The blue lines in B and C show the current slice position for this confocal scanning.

The long form of the MsrA full length protein conjugated with the eGFP tag has been studied previously in our laboratory and was found to be predominantly mitochondria located with detectable signals from the cytosol [15]. Current studies confirm the same finding with the MsrA long form-eGFP fluorescence signals (green, figure 4B); the mitochondria stained by MitoTracker (red, figure 4A) mostly overlap each other and show an orange color (figure 4G). However, the MsrA truncated form-eGFP fusion protein shows more nonspecific localization mostly in the cytosol although green fluorescent signals in mitochondria can be detected (Figure 4D, E, F and 4H). To further confirm this observation, we have generated Figure 5, a combination of three single slices of confocal scan, each from a different view angle, top view (A), upper-side view (B) and right-side view (C) on a stem cell colony with the truncated-MsrA-eGFP transfection. This permits a much clearer three dimensional configuration of the subcellular localization of the truncated protein than a single view. On the focal point of the scans (crosspoint of the horizontal green line and the vertical pink line), clearly the green fluorescent signal is excluded from nuclei stained by DAPI. Most of the green signals are not overlapping with the red mitochondria in all three view angles (arrowhead, Figure 5C) although there are some detectable colocalizations evident (arrow, Figure 5B).

Recent studies from Lee et al., [10] and Pascual et al., [11] have demonstrated the existence of two alternative promoters for the MsrA gene that encodes different isoforms of MsrA proteins that locate in mitochondria or cytosol/nuclei due to the presence or absence of a N-terminal mitochondrial signal peptide. However, studies from Kim and Gladyshev [13], using GFP fusion techniques and deletion mutagenesis have revealed other important functional domains in the MsrA peptide sequence, including sequences close to the c-terminus, that may also direct the specific locations of the protein in subcellular compartments. In addition, localization of the mitochondrial form of MsrA in the cytosol and nuclei is also noted by Kim and Gladyshev [18] in MsrA overexpression studies. Although syntheses of different isoforms with or without N-terminal signal peptide might be the optimal way for the cells to direct protein sorting, it definitely should not be ignored that the same isoform might still be able to locate to multiple cellular compartments. While we do not rule out the possibility that the altered localization pattern for the truncated protein compared to the long form is due to GFP fusion interference, it is most unlikely considering the fact the same method has been used successfully to reveal subcellular localization of MsrA in our cell lines [15]. Our studies on the truncated MsrA-eGFP fusion protein suggest a necessary domain at the c-terminal sequence for permanently docking of the protein on mitochondria. In addition to the mitochondria signal peptide, there might exist another essential domain at the c-terminal end of the full length protein, without which, the truncated proteins, are able to be sorted to the mitochondria but will eventually leak out back into the cytosol. In the deletion mutagenesis studies of Kim and Gladyshev [18], the deletion is limited only to the very end of the N-terminus or very middle, not totally overlapping the portion omitted in this truncated form which could harbor more functional domain units [18].

### Real time RT-PCR shows a different response of mRNA expression levels for the trunctated form compared to the full length MsrA

**Figure 6:**
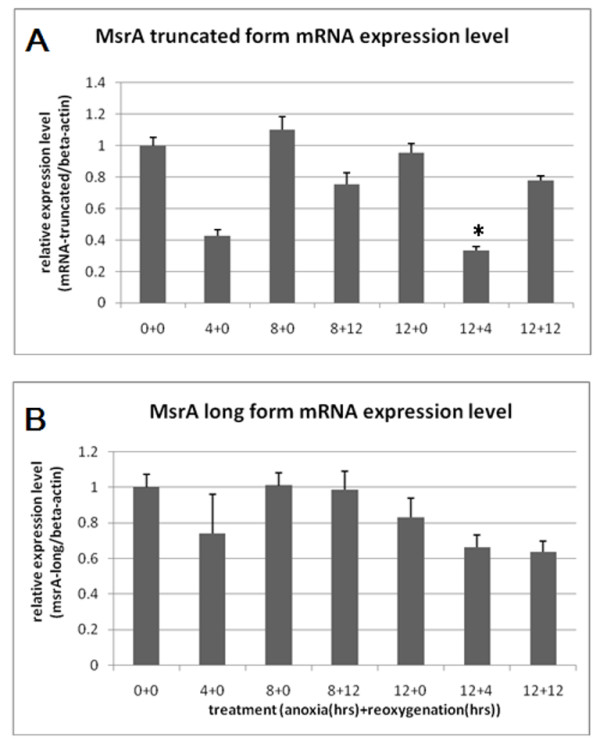
**Real time RT-PCR studies on the expression responses at the mRNA levels for the truncated MsrA (A) and long form MsrA (B) after specific hours of anoxia followed by specific hours of reoxygenation treatments**. For example, 12+4 shows 12 hours of anoxia and 4 hours of reoxygenation afterwards. *: P < 0.05 when comparing 12+4 to 12+0 and 12+12.

Our studies using real time RT-PCR on the long form MsrA expression responses to oxygen deprivation and reoxygenation show that the expression levels decrease along with longer anoxia/reoxygenation treatment combinations (Figure 6B). However, the same type of study on the truncated MsrA transcripts (Figure 6A) shows different responses compared to the long form, except that at 4 hours of anoxia treatment, both truncated form and the long form show decreases at the mRNA level which is considered as a general initial response of stem cells to oxygen deprivation as it is observed in all Msr genes as well as in other genes such as matrix metalloproteinases 2 and 9 (MMP2 and MMP9) [15]. The mRNA of the truncated form decreases most dramatically when a short reoxygenation (4 hours) was given after a long period of anoxia (12 hours) (12+4, Figure 6A). At this point, the level of reactive oxygen species (ROS) in the cells are expected to rise substantially. Expression level partially recovers after 12 hours of reoxygenation following the 12 hours of anoxia treatment (12+12), to the same level as 12 hours of reoxygenation following 8 hours of anoxia (8+12), at which point the level of reactive oxygen species might have decreased compared to the point of 12+4. Our results indicate that the truncated form of the MsrA transcript might be responsive to the cellular level of ROS. Comparing the expression levels at 12+4 and 12+12 time points for the long form and truncated MsrA mRNA, we could see that the truncated form is more sensitive to oxidative stress level changes.

## Conclusions

In summary, a c-terminal truncated form of MsrA has been cloned from mouse embryonic stem cells due to the skipping of exon 5 and subsequent frame shift in exon 6, generating a premature stop codon. The truncated protein shows a different subcellular localization and pattern of expression response to anoxia/reoxygenation treatment on the stem cells. Further study on the enzymatic activity of this peptide is needed to consider it as a functional isoform. One possibility for the necessity of having such a truncated form could be that with a smaller protein size and retained GCWFG active site, this protein might have easier access to the oxidized methionine residues on proteins with structure hindering the access for the long form protein, thus having an advantage to be evolutionarily selected and maintained. Since the truncated protein itself does not contain the c-terminal cysteines, whether the final relieving of the oxidation step needs the long form MsrA is unknown, although we did observe that heterodimers formed between long form and truncated proteins in Western blotting experiments.

## Competing interests

The authors declare that they have no competing interests.

## Authors' contributions

PJ was involved in the conception and design, provision of study materials, collection and assembly of data, data analysis and interpretation and manuscript writing for this paper. CZ participated in the conception and design, collection and assembly of data, data analysis and interpretation, manuscript writing and final approval of manuscript for publication. YJ - Provided study materials, collection of data, data analysis and interpretation and manuscript writing for the paper. KW was involved in the conception and design, financial support, administrative support, provision of study materials, collection and assembly of data, data analysis and interpretation, manuscript writing and final approval of the manuscript. XH Participated in the collection and assembly of data, data analysis and interpretation, manuscript writing and final approval of the manuscript. AK was involved in manuscript writing and data analysis. SL was involved in the conception and design, administrative support, data analysis and interpretation, manuscript writing and final approval of the manuscript. LL Oversaw the research including the conception and design, financial support, administrative support, provision of study materials, collection and assembly of data, data analysis and interpretation, manuscript writing, final approval of manuscript as well as serves as the Principal Investigator of laboratory.
